# β-Citronellol: a potential anti-inflammatory and gastro-protective agent-mechanistic insights into its modulatory effects on COX-II, 5-LOX, eNOS, and ICAM-1 pathways through in vitro, in vivo, in silico, and network pharmacology studies

**DOI:** 10.1007/s10787-024-01569-x

**Published:** 2024-09-29

**Authors:** Urooj Iqbal, Abdul Malik, Nabeela Tabassum Sial, Malik Hassan Mehmood, Shoaib Nawaz, Marios Papadakis, Dalia Fouad, Hayam Ateyya, Nermeen N. Welson, Athanasios Alexiou, Gaber El-Saber Batiha

**Affiliations:** 1 Department of Pharmacology, College of Pharmacy, University of Sargodha, Sargodha, Pakistan; 2 Institute of Pharmacy, Lahore College for Women University, Lahore, Pakistan; 3 Department of Pharmaceutical Sciences, Government College University Lahore, Lahore, Pakistan; 4 Department of Pharmacology, Faculty of Pharmaceutical Sciences, Government College University Lahore, Lahore, Pakistan; 5 The University of Lahore, Sargodha Campus, Sargodha, Pakistan; 6 Department of Surgery II, University Hospital Witten-Herdecke, University of Witten-Herdecke, Heusnerstrasse 40, 42283 Wuppertal, Germany; 7 Department of Zoology, College of Science, King Saud University, PO Box 22452, 11495 Riyadh, Saudi Arabia; 8 Department of Pharmacy Practice and Clinical Pharmacy, Faculty of Pharmacy, Future University in Egypt, Cairo, Egypt; 9 Department of Forensic Medicine and Clinical Toxicology, Faculty of Medicine, Beni-Suef University, Beni Suef, 62511 Egypt; 10 University Centre for Research and Development, Chandigarh University, Chandigarh-Ludhiana Highway, Mohali, Punjab India; 11 Department of Science and Engineering, Novel Global Community Educational Foundation, Hebersham, NSW 2770 Australia; 12 Department of Research and Development, Funogen, 11741 Athens, Greece; 13 Department of Research and Development, AFNP Med, 1030 Vienna, Austria; 14 Department of Pharmacology and Therapeutics, Faculty of Veterinary Medicine, Damanhour University, Damanhour, 22511 AlBeheira Egypt

**Keywords:** β-Citronellol, Anti-inflammatory, Gastroprotective, Molecular docking, Indomethacin, Network pharmacology

## Abstract

**Background:**

The current study aimed to evaluate the anti-inflammatory, anti-oxidant, and pronounced gastro-protective activities of β- Citronellol using in vitro*, *in vivo assays and in silico approaches.

**Methods:**

In vitro assays, denaturation of bovine serum albumin, egg protein, and human Red Blood Cells (RBCs) membrane stabilization were performed, using Piroxicam as standard. For in vivo assessment, Histamine (0.1 ml from 1% w/v) and Formaldehyde (0.1 ml from 2% v/v) were used to mediate inflammation. In silico molecular docking and network pharmacology were employed to probe the possible target genes mediating gastroprotective effect of β-Citronellol at 25, 50, and 100 mg/kg, using indomethacin-induced (25 mg/kg i.p) gastric ulcer in rats. Moreover, Gastric tissues were evaluated for morphological, histopathological, and bio-chemical analysis of PGE_2,_ COX-I, COX-II, 5-LOX, eNOS, ICAM-1, oxygen-free radical scavengers (SOD, CAT), and oxidative stress marker (MDA).

**Results:**

β*-*Citronellol prevented denaturation of proteins and RBCs membrane stabilization with maximum effect observed at 6,400 µg/mL. Citronellol decreased rat’s paw edema. Network pharmacology and docking studies revealed gastro-protective potential of Citronellol possibly mediated through arachidonic acid pathways by targeting COX-I, COX-II, PGE_2_, and 5-LOX. Citronellol reduced the ulcer indices, and histopathological changes. Further, β*-*Citronellol (50 and 100 mg/kg) increased gastric PGE_2,_ COX-1, and eNOS; while suppressing COX-2, 5-LOX and ICAM-1. Citronellol markedly enhanced the oxidative balance in isolated rat stomach tissues.

**Conclusions:**

The anti-inflammatory, anti-oxidant, and gastro-protective effects of β*-*Citronellol against indomethacin-induced gastric ulcer model in rats through mediating COX-I, COX-II, PGE_2,_ 5-LOX, eNOS, and ICAM-1 inflammatory markers.

**Graphical abstract:**

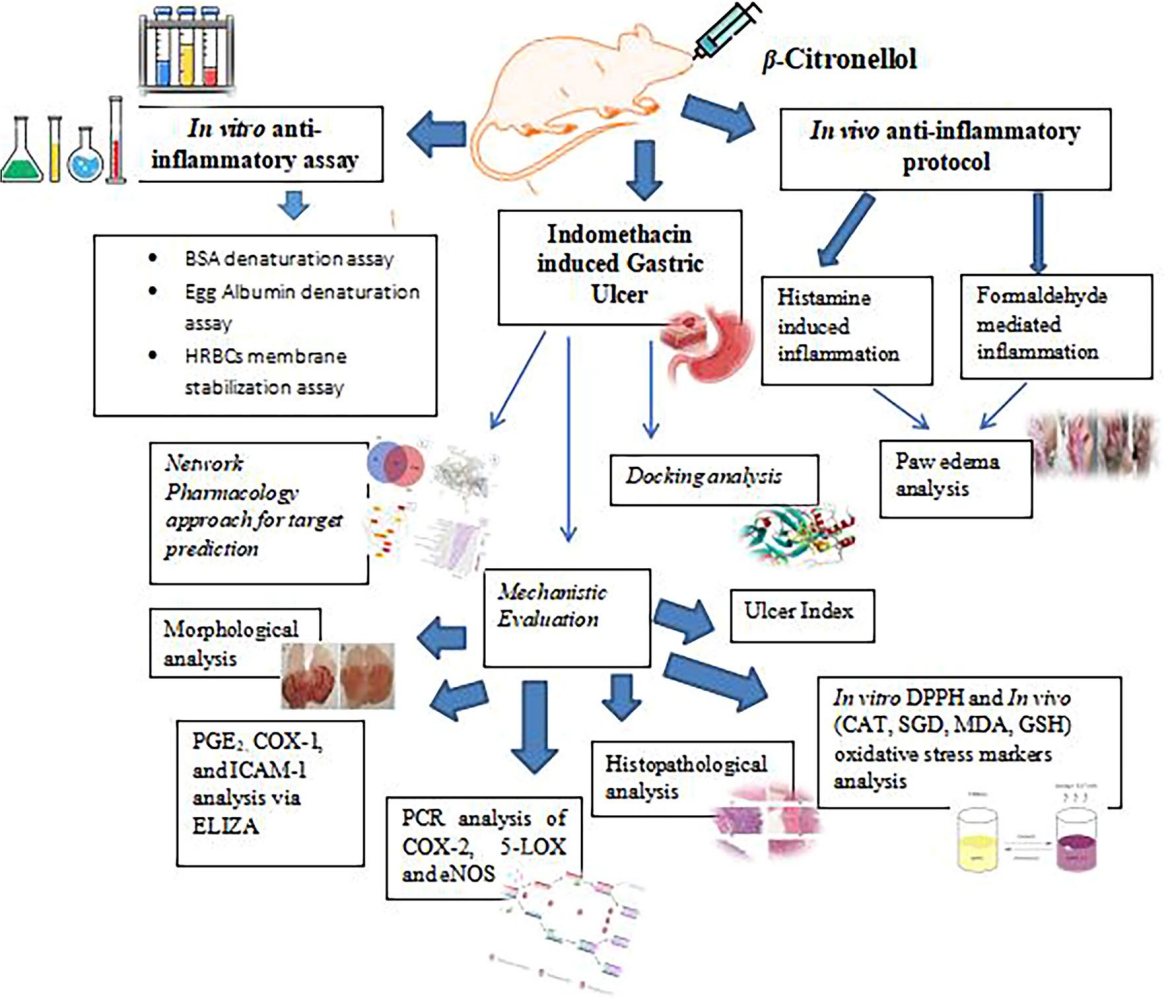

## Introduction

Inflammation is the primary response of a body against pathogenic organisms, toxic chemical substances, and physical injury to cells and tissues, which results in pain, heat, swelling, redness, and disturbances to physiological processes. In chronic inflammation, injured tissues released inflammatory mediators, including prostaglandins, histamine, nitric oxide (NO), leukotrienes, and expression of adhesion molecules, specifically intracellular adhesion molecule-1 (ICAM-1), which contribute to further inflammatory processes particularly in rheumatoid arthritis and hematological diseases (Hossain et al. 2020). Non-steroidal anti-inflammatory drugs (NSAIDs) are non-opioid medications (Panchal and Sabina 2023), used as antipyretic, anti-inflammatory, and analgesic agent and account 5–10% of all prescribed drugs worldwide. An estimated 30 million people use NSAIDs on a daily basis (Sohail et al. 2023). Indomethacin is one of the popular NSAID, for causing gastric ulcers. Gastric ulcer develops as a result of an imbalance favoring aggressive variables, such as NSAIDs, acidity, and oxidative stress, over protective factors, such as blood flow, mucus secretion, prostaglandins (PGs), nitric oxide (NO), and cell renewal (Alfadil 2024).

Usages of NSAIDs carry a number of potential adverse effects, including gastric ulceration, gastric bleeding, and restricting the gastric ulcer healing (Tahir et al. 2023). Most patients remain unable to afford the exorbitant prices of novel therapies for the management of arthritis. Moreover, standard medications utilized to treat gastric ulcer, such as proton pump inhibitors (PPIs), also exhibit a multitude of minor undesirable side effects, including headache, abdominal discomfort, constipation, and diarrhea. However, additional severe adverse effects may develop causing reduced gastric acid secretion. This facilitates the colonization of the gastrointestinal tract by pathogenic microorganisms that are typically eliminated by gastric acid. As a result, numerous infections and subsequent complications may develop (Selim et al. 2023). Thus, there is a dire need to develop safer treatment remedies (medicinal plants and their phyto-constituents) for the management of inflammation with improved gastroprotection.

**Fig. 1:**
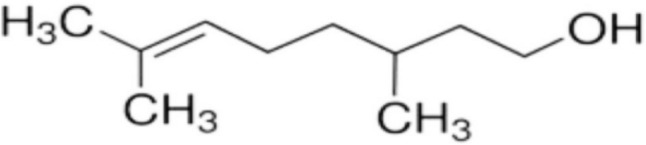
Chemical structure of Citronellol (β-Citronellol)

Several aromatic plant species, including *Cymbopogon citratus, C. winterianus*, and *Lippia alba*, contain Citronellol (β-Citronellol), a monoterpene alcohol that is an essential oil constituent (Dar et al. 2023). The chemical structure of Citronellol is illustrated in Fig. 1 (Jayaraj et al. 2022). Above-mentioned plants have been employed traditionally as anti-hypertensive and wound-healing agent. Numerous studies have documented the pharmacological properties of Citronellol, including antibacterial, anti-diabetic, antifungal, anti-hypertensive, vasodilatory, anti-oxidant, and anti-inflammatory (Santos et al. 2019). β-Citronellol inhibits nociception, thereby reduces inflammation (Brito et al. 2012). In mammary tumor tissues, Citronellol substantially downregulates cyclooxygenase-2, interleukin-6, matrix metallopeptidases-1, nuclear factor kappa-β, and tumor necrosis factor-α, whereas interleukin-10 (anti-inflammatory cytokine) is significantly up-regulated (Jayaganesh et al. 2020).

Owing to ongoing research, the current treatment option aims to reduce the gastric ulceration, one of the major adverse effects associated with the use of NSAIDs, while retaining their beneficial impact for treating pain and inflammation. Therefore, the objectives of the current evaluation are to assess prospective anti-oxidant, anti-inflammatory effects of β*-*Citronellol along with pronounced gastro-protective activity against indomethacin-induced gastric ulcer model using in vitro, in vivo*, *in silico*,* and network pharmacology approaches.

## Materials and methods

### Drugs and chemicals

β-Citronellol (C102066, Aladdin Biotech Co. Ltd. Shanghai, China), Sodium hydroxide, BSA (Bovine Serum Albumin), Tween 80, Indomethacin, Histamine, Piroxicam, and Omeprazole were obtained from Sigma-Aldrich, USA. Formaldehyde obtained from VWR, International Ltd, England. MERCK, Germany supplied the sodium citrate, di-sodium hydrogen phosphate, and dextrose. Citric acid, potassium dihydrogen phosphate, and hydrochloric acid were obtained from Riedel-de Haën, Germany. Solarbio, China, supplied the ELISA kits for Glutathione (GSH), Catalase (CAT), Superoxide Dismutase (SOD), and Malondialdehyde (MDA), while Rat ELISA kits for Prostaglandin E_2,_ COX-1, and ICAM-1 were obtained from CUSABIO, China. TRIZOL reagent (Thermo Fisher Scientific, USA), 2X SYBR PCR Master Mix (ZOKEYO, CHINA), and True cDNA Synthesis kit. All chemicals employed including diethyl ether were of analytical grade. We have confirmed the identification of compound (β-Citronellol) by GCMS and purity by HPLC.

### Animal used

Sprague–Dawley (SD) rats (150–250 g) of either sex were allocated in the animal house, located in College of Pharmacy, University of Sargodha (UOS), Sargodha. The rats were provided with standard pellet diet and tap water ad libitum. The rats were kept 12-h light/dark cycles under humidity (60–70%) and standard temperature (28 ± 2 °C). Acclimatization period of 1 week was allowed to all animals prior to the start of the experiment. The review and research methodology were approved from Biosafety and Ethical Review Committee, UOS, Sargodha, Pakistan having Certificate No. SU/ ORIC/ 3111.

### In vitro DPPH assay of β-Citronellol

DPPH (2, 2-diphenyl-1-picrylhydrazyl) in vitro scavenging protocol was used to determine anti-oxidant potential of Citronellol (Akhtar et al. 2021). Around 2 ml DPPH solution (0.1 mM) and 1 ml methanol were added to 1 ml of Citronellol/Ascorbic acid (Standard) solution of various concentrations, 3.13, 1.56, 3.13, 6.25, 12.5, 25, 50, 100, and 200 µg/ml. The mixture was allowed to incubate for 30 min. The absorbance was measured at 517 nm using methanol as a blank. Assay was carried out in thrice and percentage DPPH was calculated as represented below

$${\text{DPPH}}\;{\text{Scavenging}}\;\% {\text{age}} = \frac{{{\text{Absorbance}}\;{\text{of}}\;{\text{blank}}\left( {{\text{A}}_0 } \right)--{\text{Absorbance}}\;{\text{of}}\;{\text{sample}}\left( {{\text{A}}_1 } \right)}}{{{\text{Blank}}\;{\text{Absorbance}}\left( {{\text{A}}_0 } \right)}}*100,$$

A_0_ = Absorbance of blank, A_1_ = Absorbance of sample.

IC_50_ values were calculated to indicate antioxidant capacity, by employing linear regression analysis.

### Anti-inflammatory evaluation of β-Citronellol using in vitro methods

#### BSA protein denaturation assay

Bovine serum albumin was used to assess anti-inflammatory activity of β-Citronellol. The assay was conducted by preparing various solutions into test tubes including control, test, and product control solutions. The test solutions consist of 0.05 mL different drug concentrations (ranging from 50 to 6400 µg/mL) and 0.45 mL BSA (a 5% w/v in distilled water). Product control solutions were constituted by 0.05 mL different drug concentrations and 0.45 mL distilled water, while test control solutions comprise 0.05 mL BSA and 0.05 mL distilled water. Piroxicam was employed in as a standard. Using 1 N HCl, pH 6.3 was adjusted. After 20 min of incubation at 37 ± 2 °C, each solution was subsequently transferred to a temperature of 57 ± 2 °C for a period of 30 min. After cooling, 2.5 mL phosphate buffer solution (pH 6.3) was added in each test tube, and turbidity was determined at 660 nm by employing UV–visible spectrophotometer (Mahnashi et al. 2021). Percentage inhibition of protein denaturation was estimated by the following formula:

$$\% \;{\text{age}}\;{\text{Inhibition}} = 100 \, --\frac{{{\text{Test}}\;{\text{Solution}}\;{\text{Absorbance}}--{\text{Product}}\;{\text{Control}}\;{\text{Absorbance}}}}{{{\text{Test}}\;{\text{Control}}\;{\text{Absorbance}}}} \times 100.$$

#### Egg albumin protein denaturation assay

To evaluate inhibitory potential of β-Citronellol against denaturation of egg albumin, a reaction mixture consisting of 5 mL was prepared in a test tube: fresh egg albumin 0.2, 2 mL each drug concentration ranging from 50 to 6400 µg/mL, and phosphate buffer, 2.8 mL with 6.4 pH. Phosphate buffer, egg albumin, and distilled water constituted the control solution. For this assay, Piroxicam was utilized as the standard. After 15 min of incubation at 37 ± 2 °C, resultant mixture was allowed to heat for 5 min at 70 ± 2 °C. Following the calculation of percentage inhibition using the subsequent formula, absorbance was assessed at 660 nm subsequent to its cooling (Aslam et al. 2022).

$${\text{Percentage}}\;{\text{Inhibition}} = \frac{{({\text{Absorbance}}\;{\text{Control}}\;{\text{Solution}}--{\text{Absorbance}}\;{\text{Sample}}\;{\text{Solution}})}}{{{\text{Absorbance}}\;{\text{Control}}\;{\text{Solution}}}} \times 100.$$

#### Human red blood cells (HRBCs) membrane stabilization assay

The present study evaluated stabilizing effect of Citronellol on HRBCs membranes against heat and hypotonicity-induced membrane lysis. HRBCs’ suspension was prepared from a blood collected from an individual who had abstained from NSAID use, 14 days prior to sample collection. Equal volume of Alsver’s solution with same quantity of collected blood was centrifuged at 3000 rpm. Following the removal and threefold washing of packed cells with isotonic saline (0.85%), a 10% v/v HRBCs suspension was prepared with isotonic saline. Sample mixture consists of 1 mL phosphate buffer solution (0.15 M, pH 7.4), 1 mL from various drug concentrations (ranging from 50 to 6400 µg/mL), 2 mL hypotonic saline (0.36%), and 0.5 mL HRBCs suspension (10% v/v). 2 mL of distilled water, phosphate buffer, and HRBCs’ suspension constituted the control mixture. Piroxicam was employed as standard. After 30 min of incubation at 37 ± 2 °C, each sample was centrifuged at 3000 rpm for subsequent 20 min. Thus, supernatant absorbance was subsequently measured at 560 nm (Qasim et al. 2021).

Percentage membrane stabilization protection was estimated by:

$$\% \;{\text{age}}\;{\text{HRBCs}}\;{\text{protection}} = 100 - \left[ {\left( {{\text{Sample}}\;{\text{Absorbance}}/{\text{Control}}\;{\text{Absorbance}}} \right) \times 100} \right].$$

### Anti-inflammatory evaluation of β-Citronellol using in vivo methods

#### Histamine-induced paw edema

**Fig. 2:**
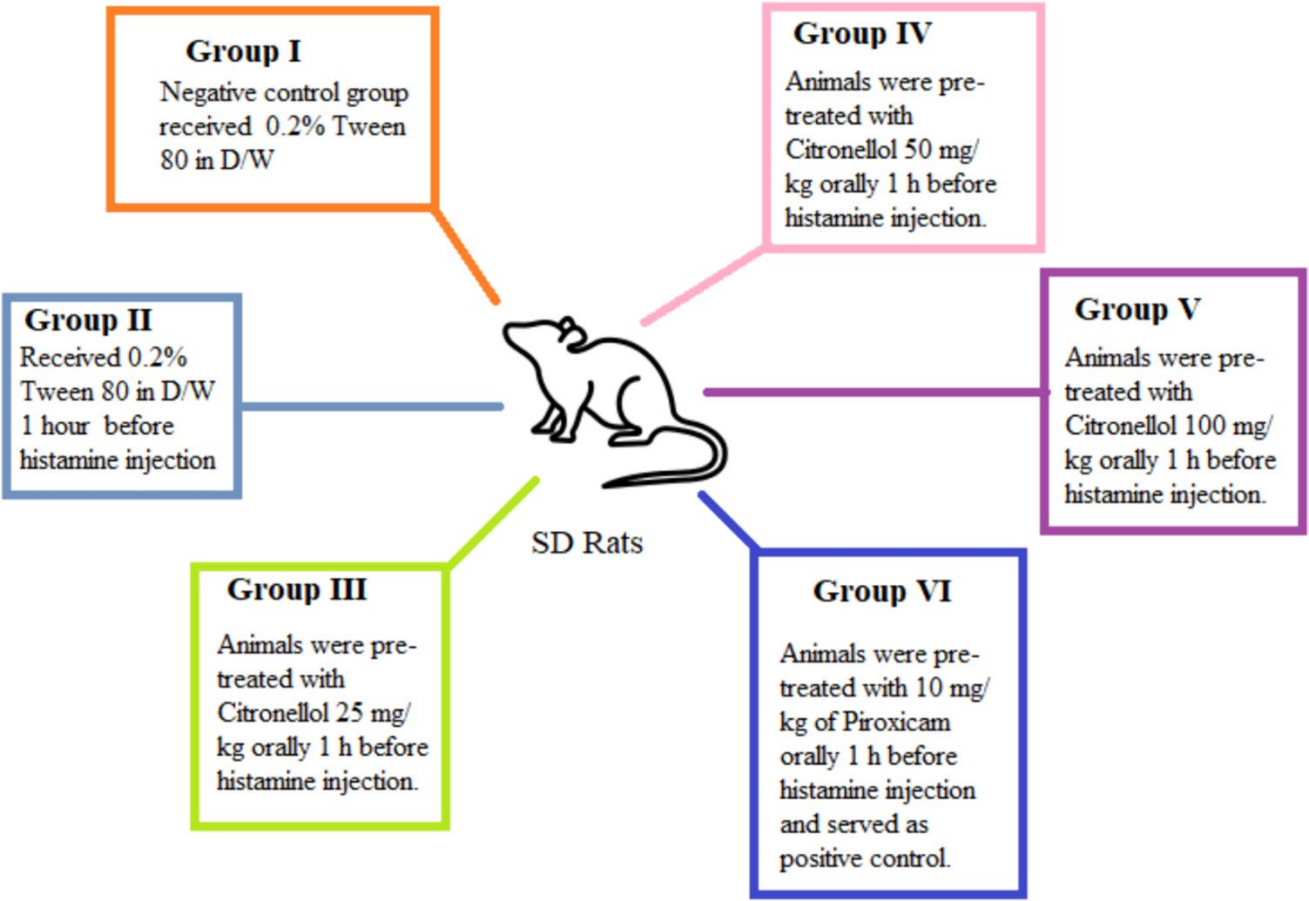
Grouping and treatment protocol

SD rats were divided into various groups containing *n* = 6 as shown in Fig. 2. Sub-plantar injection of 0.1 ml of freshly made 1% histamine caused edema in the rat’s right foot. A plethysmometer was used to assess the paw volume at baseline and then 1, 2, and 3 h following histamine injection. The inhibition of inflammation was calculated in percentage using the following formula:

$$\% {\text{paw}}\;{\text{edema}}\;{\text{inhibition}} = \frac{{{\text{Paw}}\;{\text{edema}}(Vc) - {\text{Paw}}\;{\text{edema}}\left( {Vt} \right)}}{{{\text{Paw}}\;{\text{edema}}(Vc)}} \times \, 100,$$

where *Vc* and *Vt* represent changes in paw volume in the model group and the tested groups, respectively (Osman et al. 2017; Saleem et al. 2021a, b).

#### Formaldehyde-induced inflammation

**Fig. 3:**
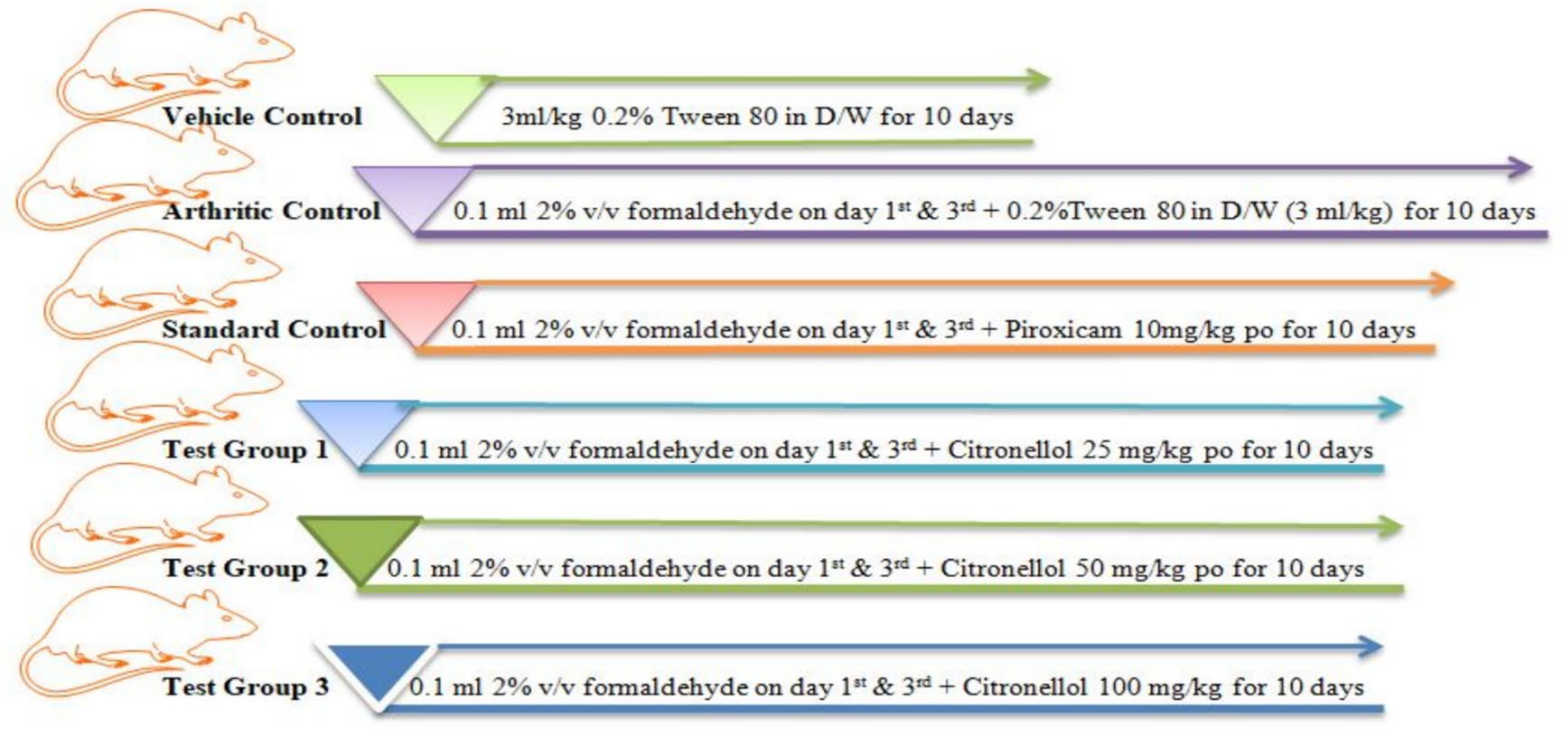
Grouping and treatment strategy protocol

Prior to experimentation, test doses were selected from previously published and reported studies that documented the nociceptive properties of Citronellol (Brito et al. 2012). Sprague–Dawley rats were utilized to assess acute non-immune arthritis, induced by injecting 0.1 mL, Formaldehyde (2% v/v) into left hind limb, 30 min after Citronellol administration using a method that had been previously documented by Akhter et al. (2021). Plethysmometer measurements of paw volume were taken on even days (two, four, six, eight, and ten). Six rats were placed in each of the various groups of SD rats as mentioned below in Fig. 3.

Paw volume inhibition percentage was calculated by the formula:

$$\% {\text{paw}}\;{\text{edema}}\;{\text{inhibition }} = \frac{{{\text{Paw}}\;{\text{edema}}\;({\text{control}}\;{\text{animals}}) - {\text{Paw}}\;{\text{edema}}({\text{treated}}\;{\text{animals}})}}{{{\text{Paw}}\;{\text{edema}}({\text{control}}\;{\text{animals}})}} \times { 1}00.$$

#### β-Citronellol ADMET analysis

The essential tool for evaluation of any compound prior to being selected as a potential drug candidate is Absorption, Distribution, Metabolism, Excretion, and Toxicity (ADMET) analysis. Online pkCSM (http://biosig.unimelb.edu.au/pkcsm/prediction) and swiss ADME (http://www.swissadme.ch/index.php) tools were utilized to investigate the pharmacokinetic and ADMET properties of the Citronellol (Hassan et al. 2021).

### Network pharmacology

#### Prediction of target for β-Citronellol and gastric ulcer (GU)

The initial step in elucidating the molecular interaction of drugs for the management of diverse ailments is the prediction of target genes. Swiss Target Prediction (http://www.swisstargetprediction.ch/) tool was utilized to forecast the targets of Citronellol, in accordance with the simplified molecular-input line-entry system (SMILES) acquired for Citronellol, retrieved from the PubChem database (https://pubchem.ncbi.nlm.nih.gov/) (Ahmed et al. 2022). By employing the keyword “Gastric Ulcer” a MeSH term associated with GU, two databases (the Online Mendelian Inheritance in Man (OMIM) (https://www.omim.org) and the GeneCards (https://www.genecards.org) were queried for GU-related targets to compile a more exhaustive database (Li et al. 2022a, b). The OMIM is an authoritative and exhaustive compilation of human genes and genetic phenotypes, while the GeneCards database entries are received from the relevant scientific literature. The Venn diagram and intersection targets of Citronellol and GU were acquired through the utilization of Evolutionary Genomics and Bioinformatics database (https://bioinformatics.psb.ugent.be/webtools/Venn/) (Liu et al. 2023).

#### Establishment of networks of protein–protein interactions (PPI)

To construct a protein–protein interaction network, the intersection targets were entered into the STRING database (https://string-db.org/) version 12.0. The organism specified in the PPI network was designated as “*Homo sapiens*” (Li et al. 2022a, b). The database provides both experimental and predicted relationship pieces of information by employing a systematic co-expression approach, automated scientific literature text mining, and examination of shared selective signals across genomes (Liu et al. 2022). For the purposes of this investigation, interactions with combined score of at least 0.4 were chosen for further analysis. Potential targets for gastric ulcer and Citronellol were imported into the Cytoscape 3.8.2. The hub nodes in the PPI network were evaluated using Cytohuba plug-in, in Cytoscape software to investigate network topological properties (Ma et al. 2022).

#### Kyoto encyclopedia of genes and genome enrichment analyses

Kyoto Encyclopedia of Genes and Genomes Enrichment Analyses (KEGG) were performed on the intersection targets using ShinyGo 0.77 databases (http://bioinformatics.sdstate.edu/go/) (Ge et al. 2020) to undertake a comprehensive analysis of the signaling pathways involved in common genes.

#### Molecular docking appraisal

β-Citronellol docking analyses were performed against a number of important proteins target, including COX-1 (PDB: 6Y3C), PGE_2_ (PDB: 7D7M), COX-2 (PDB: 5KIR), and 5-LOX (PDB: 3V98). Proteins were prepared for molecular docking using YASARA version 20.7.4, obtained from the RCSB Protein Data Bank (http://www.rcsb.org/pdb/home) (Krieger and Vriend 2014). PubChem (https://pubchem.ncbi.nlm.nih.gov/) provided the 3D structure of the ligand. Using a modified Auto Dock-Lamarckian Genetic Algorithm, YASARA software was used to determine binding energies and dissociation constants. Scores of Docking were computed using the equation mentioned

$$\Delta G=\Delta G\left(\text{van} \text{der} \text{waals}\right)+\Delta G\left(H-\text{bonding}\right)+\Delta G\left(\text{electrostatic}\right)+\Delta G\left(\text{torsional} \text{free} \text{energy}\right)+\Delta G\left(\text{desolavtion} \text{energy}\right).$$

Data regarding ligand–protein interactions were obtained using PyMol and LigPlot (Laskowski and Swindells 2011).

#### Gastric ulcer induced by indomethacin

The administration of indomethacin to rodents resulted in the development of ulcerogenic states, as previously documented by AlKreathy et al. (2020). Before oral administration of drugs, 42 SD rats (male and female) weighing 170–280 g were randomly allocated into seven groups (*n* = 6) and fasted for 16 h as under:

**Table:** 

Groups	Treatment
Group I	Vehicle control group received standard diet supplemented with the vehicle (0.2% Tween 80 in D/W)
Group II	Indomethacin control group, was administered i.p indomethacin (25 mg/kg) as a single dose, on the day of dissection
Group III	β-Citronellol 25 mg/kg orally for 3 days + i.p indomethacin (25 mg/kg) on 3rd day, 1 h after the treatment dose
Group IV	β-Citronellol 50 mg/kg orally for 3 days + i.p indomethacin (25 mg/kg) on 3rd day, 1 h after the treatment dose
Group V	β-Citronellol 100 mg/kg orally for 3 days + i.p indomethacin (25 mg/kg) on 3rd day, 1 h after the treatment dose
Group VI	100 mg/kg β-Citronellol alone for 3 days
Group VII	Pre-treated with 30 mg/kg Omeprazole orally + i.p indomethacin (25 mg/kg) on 3rd day, 1 h after the treatment dose

Rodents were then dissected 4 h after the indomethacin challenge. Animals were anaesthetized via inhalation of diethyl ether (1 mL) onto a cotton swab, subsequently sacrificed by decapitation. Rats were dissected 3 days after the experiment commenced.

#### Separation of blood samples and stomach

Following anesthesia, blood samples were drawn from the heart of the animals using blood sample tubes with anticoagulant ethylenediaminetetraacetic acid (EDTA) and allowing them to cool at room temperature (20–22 °C) for 15 min, and further were centrifuged at 3000*g* for 10–15 min. For bio-chemical analysis, the separated serum was stored at −20 °C. The surgical scalpel was employed to expose their abdominal surface. Through the greater curvature, rat’s stomachs were sectioned and rinsed with saline (0.9% NaCl). The stomach were rinsed with physiological solution once more; one portion was submerged in 10% formalin for histopathological analysis, while the other one was retained for bio-chemical analysis (Safari et al. 2023).

#### Assessment of gastric mucosal damage (ulcer index)

The stomachs of the rats were expanded and photographed using an appropriate digital camera. At a magnification of 10X, stomach mucosa was examined microscopically. Number of superficial, deep ulcers, and perforations were observed. For each rat, total numbers of ulcers were recorded. The ulcers in each rat were scored as:

**Table:** 

0	1	1.5	2	3
No ulcer	Superficial ulcers	Hemorrhagic streak	Deep ulcers	Perforations

Each group average severity score was calculated (Mostofa et al. 2017). Ulcer index (UI) was computed via the formula: (UI = UN + US + UP*10^–1^). UI = Ulcer Index, UN = Average number of ulcers per rat, US = Average severity score, and UP = Rat percentage afflicted with ulcers (de Freitas Rocha et al. 2023).

Based on the proposed method (Bawish et al. 2023), percentage ulcer protection was calculated.

$${\text{Percentage ulcer protection}} = \frac{{({\text{Control mean UI}}--{\text{Test mean UI}})}}{{\text{Control mean UI}}} \times {1}00.$$

#### Histopathological examination

Samples of representative gastric tissue were immersed in formalin solution to prepare paraffin slabs. Subsequently, sections measuring 3–4 µm in thickness were obtained using a slide microtome. Sections were subsequently stained for histological examination with eosin and hematoxylin. Specimens were then examined under a Light Microscope (X100 magnification) equipped with a digital camera. The severity of submucosal edema, mucosal hemorrhage, mucosal erosion, and submucosal inflammation was assessed by a pathologist (Seitimova et al. 2023).

#### Assessment of oxidative stress and anti-oxidant biomarkers

In gastric tissue homogenate, both oxidative stress (MDA) and anti-oxidant (SOD, CAT, and GSH) levels were measured using Eliza kits from Solarbio, China, and results were expressed in ng per mL.

#### Biochemical analysis of prostaglandin E_2_ (PGE_2_), ICAM-1 (intracellular adhesion molecule-1), and Cyclooxygenase-1 (COX-1)

Phosphate buffered saline (pH 7.4) was used for homogenizing longitudinal slices of stomach. After centrifuging the homogenate, the supernatant was collected and placed for storage at −80 °C for PGE_2_, ICAM-1, and COX-1 analyses (Bakry et al. 2023), using enzyme-linked immunosorbent assay kits: PGE_2_ (Catalogue# CSB-E07966m), ICAM-1 (Catalogue# CSB-E04576r), and COX-1 (Catalogue# CSB-E13416r) purchased from CUSABIO, China.

#### Analysis of gene expression

By employing a tri-reagent technique, ribonucleic acid was extracted from the gastric tissues. A NanoDrop spectrophotometer was used to measure and assess the amount of RNA produced. (Kamel et al. 2018). Using True cDNA Synthesis kit (ZOKEYO, CHINA), reverse transcription of the acquired RNA was done to synthesize cDNA. Briefly, the RNA was combined with 0.5 µg oligo primers, kept at 42 °C for 15 min, and then placed on ice. After 5 s of heating at 85 °C, TRUEscript HRTase was rendered inactive, and CDNA was kept at −20 °C. Using 2X SYBR qPCR Master Mix and following the manufacturer’s instructions (ZOKEYO, CHINA), the synthesized cDNA was amplified. Briefly, a 20 μL reaction mixture containing 0.5 μL of each reverse and forward primer, 10 μL of master mix, cDNA template + RNase-free water + Rox reference dye was kept in a microplate reader then in a qPCR apparatus. Cyclooxygenase-2, 5-lipoxygenase and endothelial Nitric Oxide Synthase expression levels were measured (primers shown in Table 1). To compute and standardize the levels of gene expression, glyceraldehyde-3-phosphate dehydrogenase (GAPDH) was used as a housekeeping gene. The RNA abundances were evaluated using the relative Ct method. Using formula 2(− ΔΔ*Ct*), the relative expression was computed.

**Table 1:** Primers sequence (forward and reverse) with respect to gene markers

Genes	Forward/reverse	Primer sequence	References
COX-2	Forward Reverse	5′‐AAGCCTCGTCCAGATGCTA-3′ 5′‐ATGGTGGCTGTCTTGGTAGG-3′	Bawish et al. (2023)
5-LOX	Forward Reverse	5′-CCCCCGAAGCTCCCAGTGACC-3′ 5′-TCCCGGGCCTTAGTGTTGATA-3′	Peng et al. (2011)
eNOS	Forward Reverse	5′-TTC CGG CTG CCA CCT GAT CCT AA-3′ 5′-AAC ATA TGT CCT TGC TCA AGG CA-3′	Pan et al. (2005)
GAPDH	Forward Reverse	5′-ACCACAGTCCATGCCATCAC-3′ 5′-TCCACCACCCTGTTGCTGTA-3′	Bawish et al. (2023)

### Statistical analysis

Data were measured as means ± SEM (Standard Error of Means). GraphPad Prism (version 8.0) was used to perform One-Way ANOVA (using Dunnett’s post-test) and Two-Way ANOVA (with Bonferroni post-test), while *p* < 0.05 was considered statistically significant.

## Results

### Effect of β-Citronellol on DPPH assay as an in vitro anti-oxidant

**Fig. 4:**
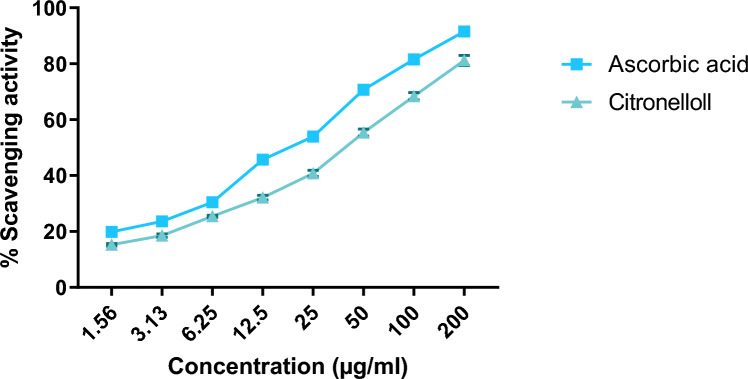
Effect of Citronellol on DPPH free radical scavenging

β-Citronellol presented the highest scavenging percentage of DPPH (81.20% ± 1.79), at 200 µg/ml concentration. Ascorbic acid has shown a significantly (*p* < 0.05) higher DPPH scavenging percentage (91.52% ± 0.75) than the Citronellol at the same concentration range, as shown in Fig. 4. The amount of sample needed to inhibit 50% of free radicals was determined using the IC50 value. The IC50 values for Citronellol and Ascorbic acid were calculated as 82.43 and 29.89 µg/ml, respectively (Table 2).

**Table 2:** IC50 value of DPPH activity of Citronellol and ascorbic acid

Sample	IC50 (µg/ml)
Citronellol	82.43
Ascorbic acid	29.89

### Suppressive impact of β-Citronellol on denaturation of bovine serum albumin

**Fig. 5:**
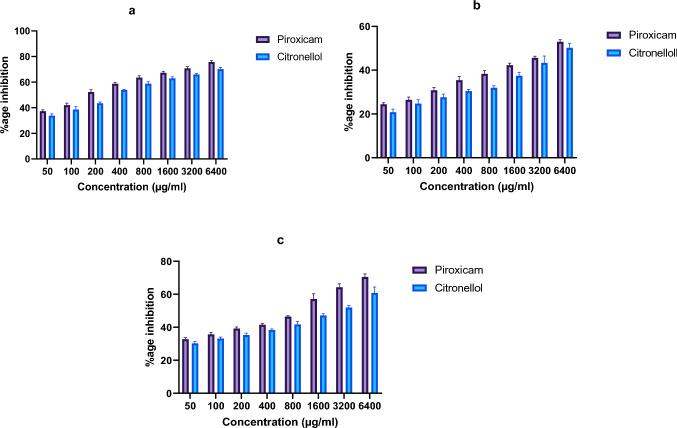
Effect of β-Citronellol on **a** bovine serum albumin denaturation and **b** egg albumin protein denaturation © HRBC membrane stabilization

Anti-denaturation assay using BSA revealed that β-Citronellol demonstrated an increasing percentage protection (33.80 ± 0.81 − 70.20 ± 0.75%) against bovine serum albumin denaturation compared to standard drug, Piroxicam (37.29 ± 0.63 − 75.73 ± 0.58%) at the concentration range from 50 to 6400 µg/mL (Fig. 5a). The highest effect was obtained at 6400 µg/mL. These findings confirm the anti-arthritic potential of Citronellol comparable to that of Piroxicam.

### Suppressive impact of β-Citronellol on egg albumin protein denaturation

β-Citronellol inhibited protein denaturation in concentration-dependent manner, induced by high temperature. The maximum effect was observed at 6400 µg/mL (50.23 ± 1.18%); in contrast to the standard drug Piroxicam (52.96 ± 0.55%) at the same concentration. Results suggest that Citronellol has potential to inhibit protein denaturation in a manner similar to that of Piroxicam (Fig. 5b).

### Protective effect of β-Citronellol on HRBCs’ membrane stabilization

The findings of the HRBCs membrane stabilization assay revealed that β-Citronellol has a positive impact on stabilizing the HRBCs membrane in a concentration-dependent manner. The maximum effect (60.79 ± 2.05%) was observed at a concentration of 6400 μg/mL. These outcomes were comparable to those of the Piroxicam, which inhibited RBCs lysis with the highest effect (70.52 ± 1.01%) at 6400 μg/mL concentration, as shown in Fig. 5c.

### Effect of β-Citronellol against histamine‑induced inflammation in rats

As demonstrated in Table 3, rats administered histamine exhibited a significant (*p* < 0.05) increase in mean hind paw volumes following 1, 2, and 3 h compared to the mean paw volume of the rats in the vehicle control group. On the other hand, the mean paw volumes of the rats pre-treated with oral β-Citronellol (25, 50, and 100 mg/kg) significantly decreased in comparison to the rats treated with histamine, showing 32.81, 43.75, and 53.21% inhibition rates, respectively (*p* < 0.05). Piroxicam inhibited the rise in paw edema by 55.46%.

**Table 3:** Impact of β-Citronellol on paw volume induced by histamine

Groups	Percentage reduction in paw volume (mL)		
	1st hour	2nd hour	3rd hour
Vehicle control	0.45 ± 0.00*** (68.88%)	0.46 ± 0.00*** (65.41%)	0.45 ± 0.00*** (64.06%)
Histamine control	1.38 ± 0.01	1.33 ± 0.02	1.28 ± 0.00
β-Citronellol 25 mg/kg	1.17 ± 0.00*** (15.21%)	0.97 ± 0.03*** (27.06%)	0.86 ± 0.00*** (32.81%)
β-Citronellol 50 mg/kg	1.15 ± 0.00*** (16.66%)	0.87 ± 0.01*** (34.58%)	0.72 ± 0.01*** (43.75%)
β-Citronellol 100 mg/kg	1.13 ± 0.00*** (18.11%)	0.77 ± 0.01*** (42.10%)	0.60 ± 0.00*** (53.21%)
Piroxicam 10 mg/Kg	1.05 ± 0.03*** (20.28%)	0.70 ± 0.00*** (47.36%)	0.57 ± 0.00*** (55.46%)

Results are presented as Means ± SEM (*n* = 6), where *p < 0.05, **p < 0.01, and ***p < 0.001, compared with disease control group using Two-way ANOVA followed by Bonferroni’s post-test.

### Effect of β-Citronellol against formaldehyde‑induced inflammation in rats

On day 10, the Formaldehyde control rats’ paw volume was considerably increased, whereas the dose-dependent reduction in paw volume was observed by β-Citronellol. A 25 mg/kg oral dosage of Citronellol demonstrated 16.59% paw volume inhibition, whereas 48.61 and 55.55% suppression of paw volume observed at 50 and 100 mg/kg, respectively. Piroxicam inhibited the rise in paw volume at 10th day, by 60.20% as shown in Table 4.

**Table 4:** Impact of β-Citronellol on paw volume induced by formaldehyde

Groups	Percentage reduction in paw volume (mL)				
	2nd Day	4th Day	6th Day	8th Day	10th Day
Vehicle control	0.70 ± 0.01*** (53.42%)	0.73 ± 0.01*** (68.88%)	0.68 ± 0.01*** (54.65%)	0.69 ± 0.01*** (53.11%)	0.68 ± 0.07*** (52.22%)
Arthritic control	1.51 ± 0.03	1.68 ± 0.01	1.51 ± 0.01	1.47 ± 0.02	1.44 ± 0.01
β-Citronellol 25 mg/kg	1.34 ± 0.01*** (11.25%)	1.37 ± 0.07*** (18.25%)	1.31 ± 0.04*** (13.33%)	1.25 ± 0.05*** (15.22%)	1.20 ± 0.04*** (16.59%)
β-Citronellol 50 mg/kg	0.77 ± 0.03*** (49.27%)	0.80 ± 0.02*** (52.02%)	0.80 ± 0.01*** (46.86%)	0.77 ± 0.02*** (47.76%)	0.74 ± 0.02*** (48.61%)
β-Citronellol 100 mg/kg	0.94 ± 0.02*** (37.54%)	0.85 ± 0.01*** (49.28%)	0.77 ± 0.01*** (48.71%)	0.70 ± 0.01*** (52.30%)	0.64 ± 0.01*** (55.55%)
Piroxicam 10 mg/Kg	0.91 ± 0.03*** (40.05%)	0.82 ± 0.02*** (51.24%)	0.72 ± 0.01*** (52.47%)	0.65 ± 0.01*** (55.68%)	0.57 ± 0.09*** (60.20%)

Results are presented as Means ± SEM (*n* = 6), where *p < 0.05 **p < 0.01 and ***p < 0.001, compared with disease control group using Two-way ANOVA followed by Bonferroni’s post-test.

### β-Citronellol ADMET analysis

The physiochemical and pharmacokinetic profile of β-Citronellol is presented in Table 5. Citronellol possesses good topological polar surface area score, best intestinal absorption, and high safety profile suggesting that it is one of the best candidates for pharmaceutical/clinical trials.

**Table 5:** Evaluation of physicochemical and pharmacokinetic parameters of β-Citronellol

Properties	Parameters	β-Citronellol
Physicochemical Properties	Molecular weight	156.27 g/mol
	Fraction Csp3	0.80
	Rotatable bonds	5
	H-bond acceptors	1
	H-bond donors	1
	TPSA^a^	20.23 Å^2^
Lipophilicity Log Po/w	Ilogp	2.72
	XLOGP3	3.91
	WLOGP	2.75
	MLOGP	2.70
	Consensus	2.92
Absorption	Intestinal absorption (human)	95.72%
	Skin Permeability	−1.682 (log Kp)
	P-glycoprotein substrate	Yes
Distribution	BBB permeability	0.538 (log BB)
	CNS permeability	−1.848 (log PS)
Metabolism	Substrate of CYP2D6	No
	Substrate of CYP3A4	No
	Inhibitor of CYP1A2	Yes
	Inhibitor of CYP2C19	No
Excretion	Total Clearance	0.492
	Renal OCT2^b^ substrate	No
Toxicity	Oral Acute Toxicity in rat	1.992 (mol/kg)
	Oral Chronic Toxicity in rat	1.934 (log mg/kg bw/day)
	Hepatotoxicity	No

^a^Topological polar surface area, ^b^Organic Cation Transporter 2

### Network pharmacology approach

#### Target prediction of β-Citronellol (CT) and Gastric Ulcer (GU)

**Fig. 6:**
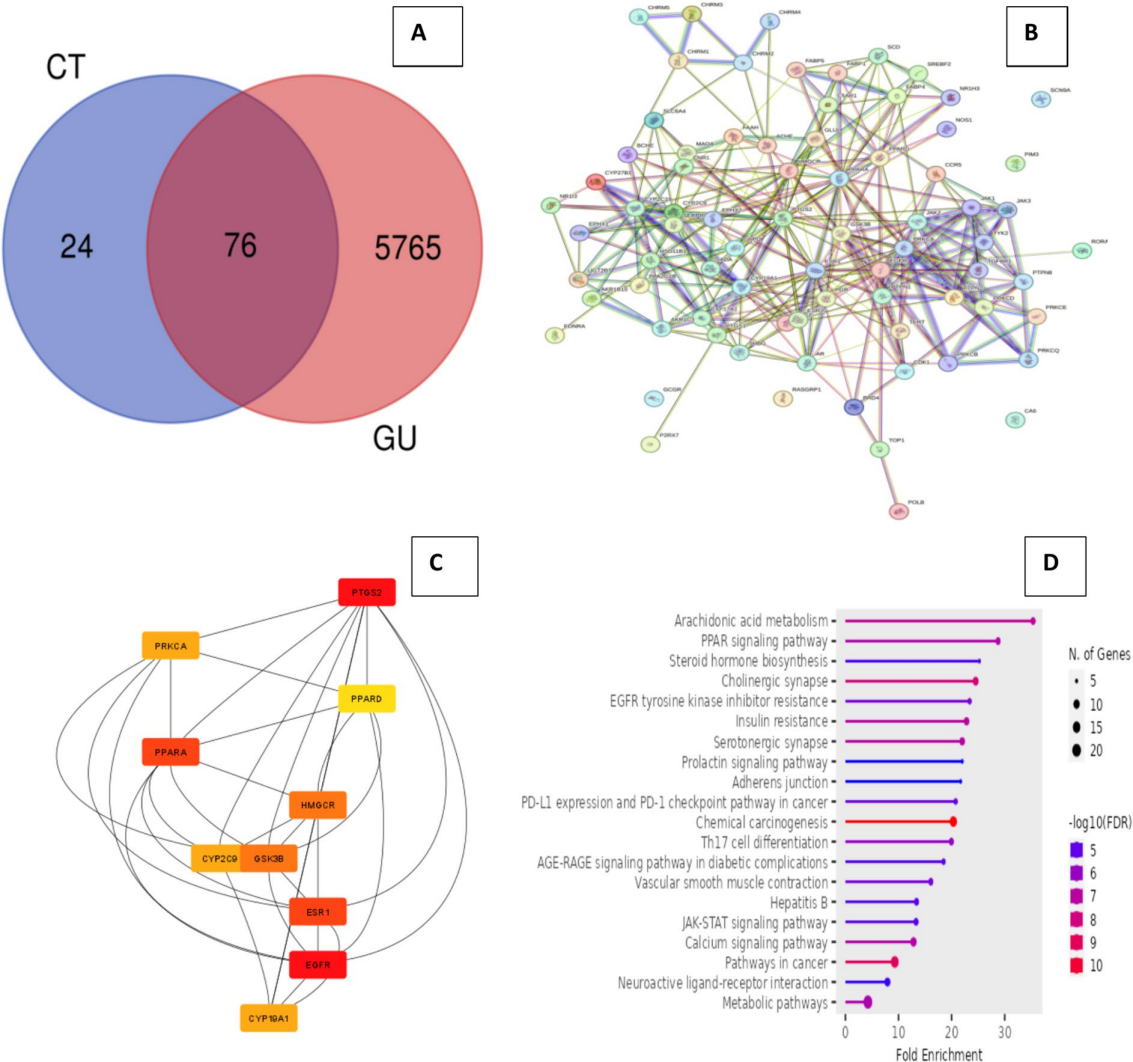
Network pharmacology of β-Citronellol in GU treatment. **A** Venn diagram of the potential targets of CT in GU treatment. **B** PPI network of 76 intersection targets. **C** According to the degree method, top ten genes in the PPI network. **D** KEGG enrichment analysis of overlapping genes

For predicting a total of 100 β-Citronellol pharmacological targets, a Swiss target prediction database was used. Likewise, a total of 6158 GU disease targets were collected from the GeneCards and OMIM databases. Using a Venn diagram, 76 intersection targets of CT and GU were found as shown in Fig. 6A.

#### Constructing protein–protein interactions (PPI) networks

The STRING database was employed to obtain the PPI network of the 76 intersection targets (Fig. 6B). The CytoHubba plug-in tool from Cytoscape 3.8.2 software was then employed to analyze the PPI network. We used the degree technique to calculate the nodes’ interactions among the 12 topological parameters in CytoHubba. All of these genes have the potential to be significant targets, because the greatest degree shows a significant association between target genes. Based on the degree-based analysis’s findings, PTGS2 (28), EGFR (28), PPARA (24), ESR1 (24), GSK3B (19), HMGCR (19), PRKCA (16), CYPC29 (16), CYP19A1 (16), and PPARD (15) were considered the top ten hub genes (Fig. 6C).

#### KEGG enrichment analyses

To find the appropriate signaling pathway linked with the anti-ulcer effect of β-Citronellol, a KEGG pathway exploration was done. The 76 intersection targets were uploaded to the ShinyGO database for KEGG enrichment analyses to explore their relationship with diseases. The metabolic pathways involved in cancer and the metabolism of arachidonic acid are the pathways with the greatest number of genes (Fig. 6D).

#### Molecular docking investigation

**Fig. 7:**
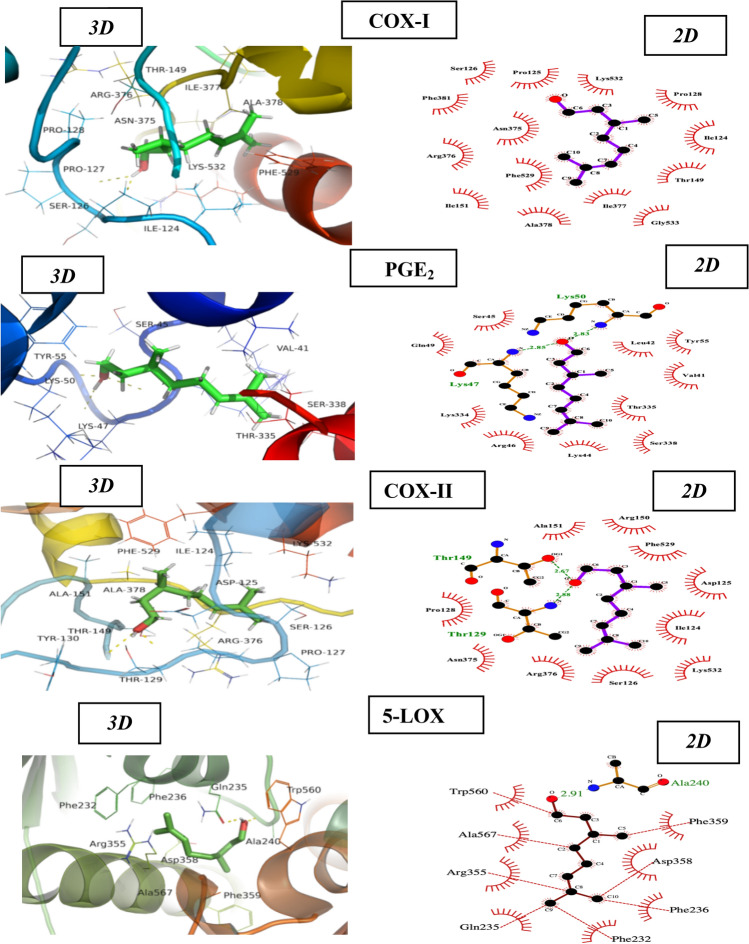
Protein pockets of Citronellol with molecular docking (2D & 3D structures)

β-Citronellol was docked against four protein targets, including COX-1, PGE_2_, COX-2, and 5-LOX, as shown in Fig. 7. Virtual screening of β-Citronellol was carried out to get ligand–protein interaction. To find the ligand–protein affinity, binding energies and dissociation constants were calculated (Table 6).

**Table 6:** Selected targets (COX-1, PGE_2,_ COX-2 & 5-LOX) of Cironellol with their Binding energies, dissociation constants and active site residues

Targets	Binding energy (Kcal/mol)	Dissociation constant (μm)	Active site residues
COX-1	−5.98	41.26	ILE 124, PRO 125, SER 126, PRO 127, PRO 128, THR 129, THR 149, ILE 151, ARG 374, ASN 375, ARG 376, ILE 377, ALA 378, PHE 381, PHE 529, LYS 532, GLY 533
PGE_2_	−5.07	19.37	VAL 41, LEU 42, LYS 44, SER 45, ARG 46, LYS 47, GLU 48, GLN 49, LYS 50, TYR 55, LYS 334, THR 335, SER 338
COX-2	−6.42	19.70	ILE 124, ASP 125, SER 126, PRO 127, PRO 128, THR 129, TYR 130, THR 149, ARG 150, ALA 151, GLN 374, ASN 375, ARG 376, ALA 378, ARG 469, PHE 470, PHE 529, LYS 532
5-LOX	−6.00	39.90	MET 231, PHE 232, GLN 235, PHE 236, GLY 239, ALA 240, ASN 241, VALA 354, ARG 355, ASP 358, PHE 359, HIS 362, TRP 560, ALA 561, ASN 566, ALA 567

β-Citronellol possessed the highest binding energies (-6.42 kcal/mol and -6.00 kcal/mol) and dissociation constants (−19.70 and 39.90 µM, respectively) against COX-II and 5-LOX, respectively.

#### Effect of β-Citronellol pre-treatment on gastric mucosa

**Fig. 8:**
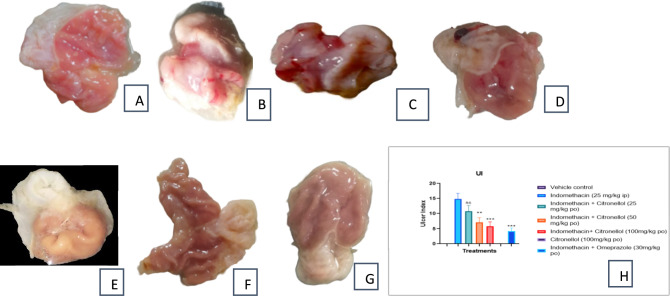
Rat’s stomach macroscopic photographs; **A** normal mucosa manifested by control group; **B** severely hemorrhagic mucosal layer by Indomethacin-exposed group; β-Citronellol-pre-treated (25, 50 and 100 mg/kg) groups (**C**, **D**, and **E** respectively) showing less severe bloody streaks than indomethacin-exposed group; **F** Citronellol 100 mg/kg-alone treated group, presenting about normal mucosa with minor injuries, **G** Omeprazole-pre-treated group (30 mg/kg) demonstrated, protected mucosal layer (**H**) UI = UN + US + UP*10^–1^

The control group stomach tissue depicts normal mucosa and no damage (Fig. 8A). On the other hand, indomethacin-exposed group showed lesions with bloody streaks that ranged in length from 0.5 to 4.5 mm (Fig. 8B), which were verified by a significantly elevated ulcer index (Fig. 8H). Rats pre-treated with Citronellol (25 mg/kg) revealed significant bloody streaks with just 27.51% ulcer protection (Fig. 8C), while 50 and 100 mg/kg of Citronellol pre-treated rats showed minor injuries manifested by congestion of the superficial blood vessels, with significant reduction in ulcer index displaying 51.71 and 60.89% protection from ulcer compared to only indomethacin-exposed group (Fig. 8D & E, respectively). Citronellol (100 mg/kg) alone administered group demonstrated typical morphology of stomach tissue evidenced by zero ulcer index and 100% protection from ulcer (Fig. 8F). With Omeprazole pre-treatment, the mucosal layer was well protected (71.20%), from ulcers with no significant difference in the ulcer index among vehicle control and the Omeprazole-pre-treated group (Fig. 8G).

#### Histopathological observations

**Fig. 9:**
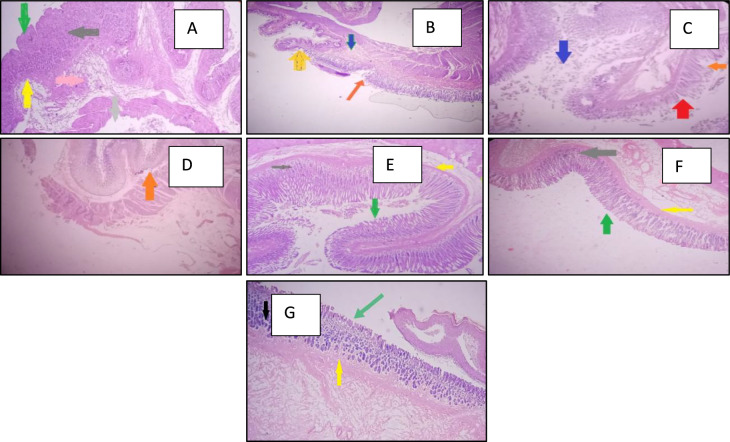
The impact of β-Citronellol pre-treatment on the histopathological alterations in gastric tissue caused by indomethacin Group (**A**): Vehicle control group. Group (**B**): Indomethacin-exposed group. Groups **C**, **D**, and **E**, represent Citronellol pre-treatment with 25, 50, and 100 mg/kg respectively; Group (**F**) Citronellol 100 mg/kg alone treated group. Group (**G**) represent Omeprazole 30 mg/kg. Green arrow shows intact mucosa • Yellow arrow depicts muscularis mucosa • Black arrow shows gastric glands • Pink arrow shows blood vessels • Red arrow represent necrotic zone • Orange arrows shows ulcerated mucosa and Blue arrow shows inflammatory cells

The control group’s histopathology analysis indicated a normal stomach histological architecture, as seen in Fig. 9A. Indomethacin-exposed group, on the other hand, displayed reduced mucosal thickness and damaged mucosal layer lining epithelium associated with ulceration, necrosis, and blood vessel congestion (Fig. 9B). In a dose-related manner, the mucosal layer’s epithelium lining of β-Citronellol pre-treatment groups receiving 25, 50, and 100 mg/kg (Fig. 9C–E, respectively) had shown improvement in epithelium lining and little congestion. Likewise, alone Citronellol-treated group (Fig. 9F) displayed no significant histopathological alterations, difference from the control group, while group that received Omeprazole pre-treatment (Fig. 9G) displayed normal gastric tissue histology.

#### Effect of β-Citronellol on anti-oxidant and oxidative stress biomarkers

**Fig. 10:**
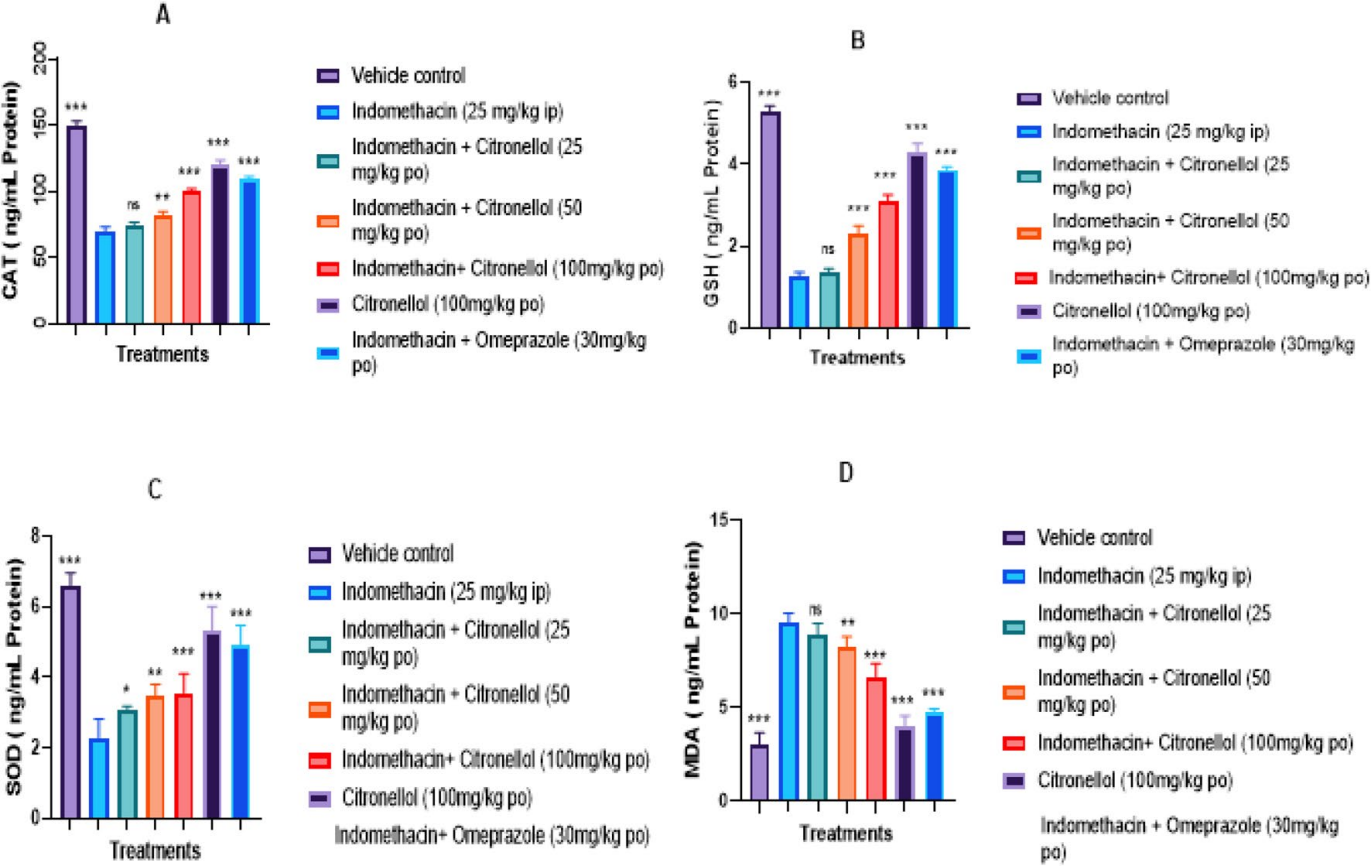
Impact of pre-treatment with β-Citronellol on indicators of oxidative stress: **A** Concentration of CAT, **B** Content of GSH, **C** Concentration of SOD, and **D** MDA activity in in rats with stomach ulcers caused by indomethacin. Using One-way ANOVA followed by Dunnett’s post*-*test, data are illustrated as mean ± S.D. (*n* = 6)

Concentrations of SOD (2.29 ± 0.21 ng/mL), CAT (69.53 ± 3.31 ng/mL), and GSH (1.27 ± 0.10 ng/mL) were significantly (*p* < 0.001) lowered in the groups exposed to indomethacin. In contrast, the concentrations of the aforementioned biomarkers increased in the groups pre-treated with Citronellol (50 and 100 mg/kg) and Omeprazole (30 mg/kg). It was observed that Citronellol (100 mg/kg)-alone treated rats demonstrated no significant difference, compared to vehicle control opposing to Citronellol (25 mg/kg), displayed no noteworthy difference in comparison to indomethacin-exposed group, as presented in Fig. 10A–C, respectively. MDA concentration in indomethacin-exposed rats (9.48 ± 0.21 ng/mL) was significantly elevated (*p* < 0.001), whereas MDA level was significantly reduced by Citronellol (50 and 100 mg/kg) pre-treated groups in a dose-related manner, and Omeprazole (30 mg/kg) pre-treated rats. However, as seen in Fig. 10D, Citronellol pre-treated group at a dose of 25 mg/kg (8.89 ± 0.23 ng/mL) did not significantly differ from the indomethacin-exposed group, whereas the group treated with Citronellol alone at a dose of 100 mg/kg (3.99 ± 0.21 ng/mL) did not significantly differ from that of vehicle control group (2.97 ± 0.21 ng/mL).

#### Impact of β-Citronellol pre-treatment on PGE_2,_ COX-1, and ICAM-1concentration

**Fig. 11:**
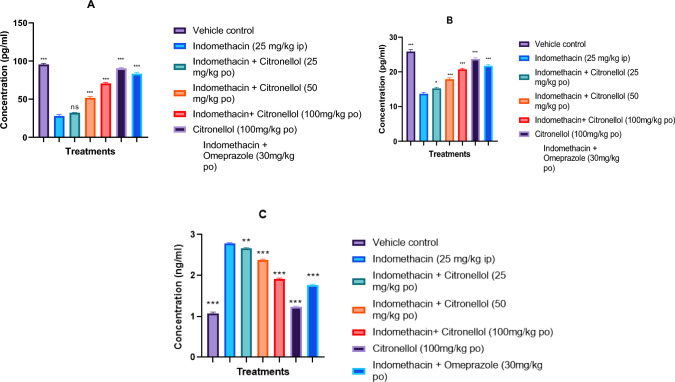
Impact of β-Citronellol pre-treatment on: **A** PGE_2_, **B** COX-1, and **C** ICAM-1 concentration in gastric ulcer caused by indomethacin in rats. Data are presented as mean ± S.E.M (*n* = 6), while *p* < 0.05 using One-Way Analysis of Variance followed by Dunnett’s post-test

The PGE_2_ and COX-1 concentration in the indomethacin-exposed group was considerably (*p* < 0.001) lower compared to vehicle control group, as Fig. 11A, B illustrates. In contrast to the indomethacin-exposed group (27.86 ± 1.86 and 13.68 ± 0.45 pg/mL), the pre-treatment of Citronellol 50 mg/kg (51.63 ± 2.12 and 17.89 ± 0.38 pg/mL), 100 mg/kg (70.57 ± 1.64 and 20.74 ± 0.29 pg/mL) and Omeprazole 30 mg/kg (83.39 ± 2.01 and 21.79 ± 0.31 pg/mL) markedly (*p* < 0.001) increased PGE_2_ and COX-1 levels, respectively. However, in case of PGE_2,_ there was no discernible difference between the group that had indomethacin exposure and the one that received Citronellol pre-treatment at a dose of 25 mg/kg (32.04 ± 0.48 pg/mL). PGE_2_ and COX-1 level did not differ significantly between the rats treated with Citronellol alone at a dose of 100 mg/kg (90.52 ± 1.14 and 23.73 ± 0.35) and the vehicle control group (95.49 ± 1.45 and 25.86 ± 0.57 pg/mL).

On the other hand, as demonstrated in Fig. 11C, exposure to indomethacin (2.78 ± 0.01 ng/mL) resulted in a noteworthy (*p* < 0.001) increase in the concentration of ICAM-1 in stomach tissue homogenate compared to vehicle control group (1.06 ± 0.03 pg/mL). In contrast, Omeprazole-pre-treated group (1.76 ± 0.01 ng/mL) showed a substantial (*p* < 0.001) decrease in ICAM-1 concentration in comparison to the indomethacin-exposed group. Furthermore, pre-treatment with Citronellol 25 mg/kg (2.66 ± 0.01 ng/mL), 50 mg/kg (2.38 ± 0.01 ng/mL), and 100 mg/kg (1.91 ± 0.01 ng/mL) resulted in dose-related decrease in ICAM-1 concentration that was statistically significant (*p* < 0.001) compared to indomethacin control group.

#### Effect of β-Citronellol pre-treatment on gene expression levels (COX-2, 5-LOX, and eNOS)

**Fig. 12:**
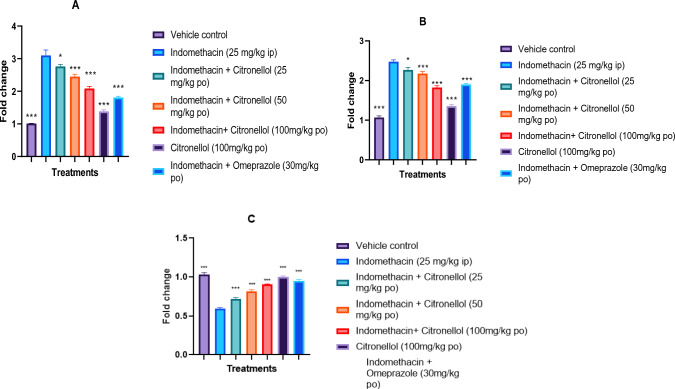
Effect of β-Citronellol pre-treatment on: **A** COX-2 level, **B** 5-LOX, and **C** eNOS level in gastric ulcer induced by indomethacin in SD rats. Data are presented as mean ± S.E.M (*n* = 6), while *p* < 0.05 using One-way analysis of variance followed by Dunnett’s post-test

As shown in Fig. 12A, B, in comparison to the vehicle control group, the indomethacin-exposed group displayed a significantly higher (*p* < 0.001) COX-2 and 5-LOX concentration. While, pre-treatment of Citronellol at 50 mg/kg (2.45 ± 0.06 and 2.17 ± 0.05), 100 mg/kg (2.08 ± 0.06 and 1.82 ± 0.04) and Omeprazole 30 mg/kg (1.81 ± 0.03 and 1.90 ± 0.01) significantly (*p* < 0.001) decreased COX-2 and 5-LOX levels, compared to indomethacin-exposed group (3.10 ± 0.16 and 2.47 ± 0.04), respectively. However, rats administered 100 mg/kg of Citronellol alone showed no apparent variation in COX-2 (1.38 ± 0.04) and 5- LOX (1.35 ± 0.03) levels when compared with the vehicle control groups (1.01 ± 0.00 and 1.06 ± 0.03) respectively.

On the other hand, as demonstrated in Fig. 12C, exposure to indomethacin resulted in a significant (*p* < 0.001) decrease in the concentration of eNOS in stomach tissue homogenate when compared to the vehicle control. In contrast, eNOS concentration was significantly (*p* < 0.001) higher in Omeprazole 30 mg/kg (0.95 ± 0.01) treated group compared to indomethacin (0.59 ± 0.01) group. Citronellol pre-treatment at doses of 25 mg/kg (0.71 ± 0.02), 50 mg/kg (0.81 ± 0.01), and 100 mg/kg (0.90 ± 0.00) also resulted in a dose-related, substantial (*p* < 0.001) increase in eNOS concentration when compared to the indomethacin-exposed group.

## Discussion

Non-steroidal anti-inflammatory drugs are being used worldwide for the management of pyrexia, analgesia, and inflammation. Chronic use of these medicines is associated with adverse effects including free radicals formation and gastric injuries (Mahmoud et al. 2023). Several medications, including Antacids, Proton pump inhibitors, Histamine H_2_ receptor blockers, Anticholinergics, Cytoprotective drugs, and Antibiotics for ulcer caused by *Helicobacter pylori*, are being used to treat peptic ulcers (Awaad et al. 2013). Unfortunately, present treatment options have potential to cause a multitude of adverse effects, impede the healing phase, and enhance recurrence, which place a substantial financial strain on patients as well as healthcare system across the globe (Byrne et al. 2018). Therefore, it is vital to investigate safer and effective natural compounds to treat inflammatory condition such as arthritis with additional gastro-protective potential. In this regard, naturally abundant secondary metabolites possess diverse biological activities (Roy et al. 2023). β-Citronellol (Citronellol) has been found in different medicinal plants having anti-inflammatory properties (Boukhatem et al. 2020). Objective of a present research was to evaluate the pharmacological activity of Citronellol as anti-inflammatory agent by the use of various in vitro and in vivo models with network pharmacology approach to expose the genes that can be targeted by β-Citronellol for treating gastric ulcer.

The therapeutic agent possessing anti-inflammatory potential also possesses anti-oxidant activity, so the anti-oxidant potential of Citronellol was assessed via DPPH free radical scavenging assay. When DPPH, a solid, dark-colored crystalline molecule, is converted to DPPH-H, it becomes colorless or pale yellow hence neutralized the DPPH in the presence of Citronellol (Baliyan et al. 2022). Compounds with IC50 values between 50 and 100 mg/mL are considered to have moderate anti-oxidant activity, while test compounds with IC50 value between 10 and 50 mg/mL are thought to have potent anti-oxidant properties (Jadid et al. 2017). In this case, Citronellol demonstrated intermediate anti-oxidant activity.

Heat, acid, base, and extrinsic stress can cause protein denaturation, which disrupts the primary, secondary, tertiary, and quaternary structures of proteins. Modifications in disulfide, hydrophobic, hydrogen, and electrostatic bonds are the primary mechanisms responsible for protein denaturation. According to a study conducted by Alamgeer et al. (2017), auto-antigens produced as a result of in vivo protein denaturation in inflammatory condition like rheumatic disease activate the immune system and cause inflammation. Citronellol has shown encouraging protein denaturation inhibition in the current study. When the membrane around the lysosomes ruptures during an inflammatory phase, chemicals, and proteases, hydrolyzing enzymes are released (Bag et al. 2013) that are involved in inflammation through digestion of cellular proteins (Saqib et al. 2021).

To prevent lysosomal contents release (phospholipase A2, bactericidal enzymes, and proteases), the lysosomal membrane must be stabilized. Studies have shown that non-steroidal anti-inflammatory drugs (NSAIDs) prevent the lysosome from rupturing and releasing its contents, stabilizing the lysosomal membrane. Red blood cells (RBCs) were employed in studies, because their membranes mimic that of lysosomal membranes. Hemoglobin is released when the membrane of erythrocytes ruptures due to hypotonic stress or heat (Saleem et al. 2020). Outcomes of the study demonstrated Citronellol prevented HRBCs rupture and membrane hemolysis. Hence, exhibit strong anti-inflammatory and membrane-stabilizing potential.

In the current investigation, rats given sub-plantar histamine injections showed a substantial rise in the hind paw volumes after 1, 2, and 3 h when compared to the paw volumes of vehicle control rats. Leukocyte migration, connective tissue proliferation, and increased vascular permeability are the three phases of inflammation. Swelling is the first sign of inflammation. In the presence of histamine, endothelial cells release prostaglandins and neuropeptides that lead to hyperalgesia and other pro-inflammatory effects (Osman et al. 2017), which were minimized by various doses of Citronellol.

The Formaldehyde-induced inflammatory model is an acute model used to investigate the anti-inflammatory properties of Citronellol. When Formaldehyde is injected into a rat’s paw, it denatures the protein; causing inflammation, localized discomfort, and triggers an immune response (Al-Joufi et al. 2024). Formaldehyde induces arthritis by a two-phase mechanism that includes an inflammatory phase and a neurogenic phase. Bradykinin, histamine, nitric oxide, prostaglandins, and serotonin are the chemical mediators released by injured cells that mediate the inflammatory phase and result in edema. Substance P and bradykinin mediate direct nociceptor stimulation in neurogenic phase. Anti-inflammatory drugs have little effect on the neurogenic phase (Saleem et al. 2021a, b). Drugs acting on the central nervous system (CNS) consistently suppress both stages, whereas peripherally acting agents act only on inflammatory phase (Farrukh et al. 2022). In the present investigation, paw edema in rats caused by Formaldehyde injection in the sub-planter region on even days (2, 4, 6, 8, and 10) was considerably reduced by oral administration Citronellol 25, 50, and 100 mg/kg. Citronellol at 100 mg/kg dramatically decreased carrageenan-induced paw inflammation, according to a previous study (Xie et al. 2021). Further, a recent study showed that administration of Citronellol significantly decreased paw edema in CFA-induced arthritis murine model (Dar et al. 2023). In line with the previous study, inhibition of protein denaturation and reduced inflammatory mediators release could be the basis for the anti-inflammatory effect of Citronellol.

**Fig. 13:**
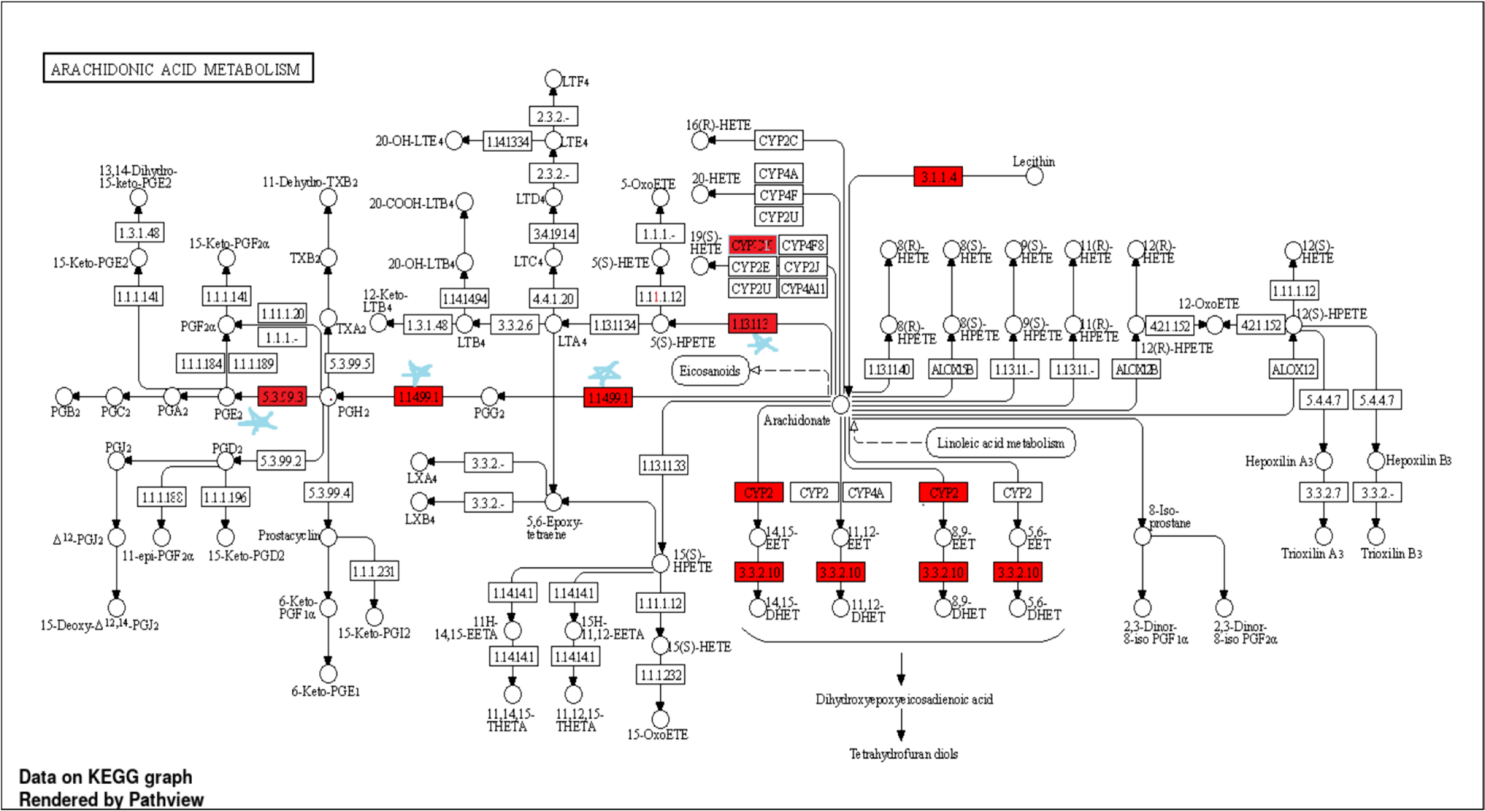
Arachidonic acid metabolism pathway; red highlighted genes are related to gastric ulcer, whereas (*) genes are targeted in the in vivo experimental study

An integrative in silico method, network pharmacology aims to create a “protein-compound/disease-gene” network to uncover the mechanisms behind the complementary therapeutic effects of conventional drugs. As a result of this development, the concept of therapy has changed from one of “one target, one drug” to one of “network target, multiple component therapeutics” (Chandran et al. 2017). Network pharmacology-based prediction approach was employed to identify the potential target genes that Citronellol may focus on for treating gastric ulcer as one of the common side effects of medications used to manage rheumatoid arthritis. Network pharmacology analysis presented 76 intersecting genes between Citronellol and targets related to gastric ulcer. PPI and KEGG pathways were used to further examine these genes. Some significantly enriched pathways among the top ten pathways in the KEGG study targeted by overlapping genes include arachidonic acid pathway, metabolic pathway, and PPAR signaling pathway. Arachidonic acid pathway was of particular interest because of an evident role in gastric ulcer as illustrated in Fig. 13.

A computational method known as “molecular docking” forecasts the affinity of ligands toward receptor proteins (Sial et al. 2024). The principal aim of docking is to acquire a ligand–receptor complex that has been optimized in terms of conformation and with the specific purpose of reducing the binding free energy. Proteins (enzymes) can be significantly inhibited by ligands (drugs) that possess a low binding energy (Morris and Lim-Wilby 2008). The current study employed molecular docking to predict how ligands (drugs) would interact with proteins (enzymes) and to determine the preferred binding mode of a ligand. In silico studies of Citronellol against protein targets including COX-1 & 2, PGE_2_ and 5-LOX shows considerable inhibition. The above-mentioned protein targets are important components in the arachidonic acid pathway involving inflammation.

Membrane phospholipids contain arachidonic acid, the substrate for eicosanoids synthesis. Eicosanoids are oxidized derivatives of 20 carbon polyunsaturated fatty acids, formed by cyclooxygenase, lipoxygenase, and cytochrome P-450 pathways. The enzyme cyclooxygenase (COX) contains two isoforms, COX-1 and COX-2, act as cyclooxygenase and peroxidase. By adding two oxygen molecules to arachidonate, these enzymes change it into prostaglandin G_2_, which is then reduced to prostaglandin H_2_. An assortment of prostaglandins, with both autocrine and paracrine activities, including as prostaglandin E_2_, prostacyclin, and thromboxane, are produced from the unstable metabolite prostaglandin H_2_. The hydroxy fatty acid, lipoxins, and leukotrienes are produced by the lipoxygenase pathway. The cytochrome P-450 pathway produces derivatives of epoxy and omega (Wang et al. 2021). To assess leukocyte activation following gastric injury, other elements of the inflammatory process, such as adhesion molecule expression, i.e., intracellular adhesion molecule-1 (ICAM-1), are also essential. Similarly, Nitric oxide (NO) is crucial to both the inflammatory response and host defense. Endothelial nitric oxide synthase (eNOS) produces NO, which is crucial for the healing of stomach ulcers (Pan et al. 2005). Therefore, by focusing on the eicosanoids, ICAM-1 and eNOS pathways, we studied the gastro-protective potential of Citronellol against indomethacin-induced stomach ulcers in rats.

A commonly used NSAIDs-induced stomach ulcer model was employed in a study. Indomethacin thought to be the most potent NSAID that causes ulcer in people (Suleyman et al. 2010). In the gastric mucosa of diseased control group, the significant elevation of inflammatory cells, peroxidation of lipids, and reactive oxygen species generation shown indomethacin is ulcerogenic to the stomach mucosa (Martin and Wallace 2006). Current research suggests that Citronellol might prevent gastric ulcer caused by NSAIDs (Indomethacin) by interfering with the COX and LOX pathways, which are linked to oxidative stress, inflammation, and ulcerative potential. Omeprazole is widely used in clinical practice to prevent stomach ulcer, and has been used as a reference drug in many studies for preventive effects of gastroenteritis/ gastric ulcer (Sidahmed et al. 2013).

In the present investigation, rat’s stomach tissues revealed severe morphological damage due to indomethacin administration. However, in contrast to indomethacin, pre-treatment of Citronellol (50 and 100 mg/kg) has shown fewer injuries and a significant decrease in the stomach ulcer index. Citronellol 100 mg/kg demonstrated no major changes in histopathology. Histopathological examination of stomach tissues of the ulcerated rats demonstrated hemorrhagic lesions that deeply pierced the mucosa as well as a notable erosion of the stomach mucosa. Additionally, the ulcerated group had pathological signs in the submucosal layer, including substantial edema and leukocyte infiltration (De Araújo et al. 2018).

Increased concentrations of reactive oxygen species (hydroxyl radicals, hydrogen peroxide, and superoxide anions) are responsible for gastric ulcers and gastric hemorrhage (Bandyopadhyay et al. 2002). Intracellular anti-oxidant enzymes, including catalase, can mitigate the harmful effects of reactive oxygen species (ROS) by protecting against their side effects. As an anti-oxidant enzyme, SOD shields biological systems from superoxide free radicals. GSH, on the other hand, neutralizes ROS to prevent tissue damage. Furthermore, oxidative stress has the ability to increase lipid peroxidation and MDA (a resultant byproduct), commonly employed as a measure of lipid peroxidation. The present study displayed increased oxidative stress resulting from indomethacin administration, as evidenced by a noteworthy elevation in MDA concentration, a measure of lipid peroxidation and a reduction in endogenous antioxidants levels including GSH, SOD, and CAT in stomach tissue. Aforementioned outcomes are in line with the previous study that demonstrated indomethacin is essential for ROS production and gastric mucosal apoptosis (AlKreathy et al. 2020). Conversely, it was observed that pre-treatment with Citronellol might effectively mitigate adverse effects induced by reactive oxygen species in a dose-dependent pattern. These outcomes are consistent with prior research, which demonstrated that Citronellol prevented lipid peroxidation and oxidative stress and increased levels of anti-oxidant enzymes (GSH, SOD, and catalase) (Jayaraj et al. 2022). Notably, Omeprazole exhibits substantial anti-oxidant activity in vitro by effectively removing hypochlorous acid at acidic pH levels that simulate the conditions encountered in the stomach. Further study has shown that Omeprazole effectively inhibits the production of hydroxyl radicals induced by stress, thereby preventing oxidative injury to gastric tissue (Biswas et al. 2003).

Cyclooxygenase (COX) enzyme inhibition is the key step involved in the mechanism of drugs like NSAID. The inducible COX-2 enzyme is involved in acute or chronic inflammation, while COX-1 is primarily involved in physiological processes. Leukotrienes also play role in inflammatory processes. Arachidonic acid’s conversion to 5-LOX can be shifted by COX-1 and COX-2 inhibitors inhibiting COX pathways. The 5-LOX products, such as Leukotriene (C4-E4), 5-hydroperoxyeicosatetraenoic acid, and 5-hydroxyeicosatetraenoic acid, are raised in tissue due to this shunt-like process. This mechanism is critical in inducing inflammation, which in turn leads to GI injury. When compared to the traditional anti-inflammatory medications, the development of dual inhibitors of COX/5-LOX is anticipated to improve gastric tolerance while exhibiting strong anti-inflammatory action (Huang et al. 2019). Consistent with the earlier research (AlKreathy et al. 2020), gastric damage by indomethacin was primarily due to inhibition of COX-1 enzyme, leading to the suppression of PGE_2_ production with loss of gastric protection. Further increase expression of COX-2 and 5-LOX by indomethacin modulates inflammatory responses as evidenced by the current study and consistent with previously published data (Tries et al. 2002). These actions were prevented by β-Citronellol pre-treatment.

Numerous cell types, including leukocytes and endothelial cells, express ICAM-1, a cell adhesion protein that resembles immunoglobulin (Ig). It helps leukocytes migrate transendothelially to inflamed regions. In response to inflammatory stimuli (Indomethacin), ICAM-1 is up-regulated in the vascular endothelium. This, in turn, controls leukocyte recruitment and adherence to the endothelium in the early phases of vascular inflammation (Asaad and Mostafa 2022). Pretreatment of Citronellol decreased the ICAM-1 level in indomethacin-exposed gastric mucosa, results are inline with the previously reported study (Hiratsuka et al. 2005). On the other hand, nitric oxide, which is produced from eNOS, is an essential mediator that speeds up the healing of gastric ulcer, because it preserves the integrity of the gastric epithelium, controls blood flow to the mucosa, and promotes the production and secretion of gastric mucus. By increasing eNOS mRNA expression, which was down-regulated by indomethacin exposure, β-citronellol can boost NO production (Dejban et al. 2020).

## Conclusions

Outcomes of the current study demonstrated a prominent anti-oxidant and anti-inflammatory effect of β-Citronellol evident by its protective effects on oxidative stress (MDA) and anti-oxidant biomarkers (SOD, GSH, and CAT) levels and against histamine and formaldehyde-induced inflammation in rats. The gastro-protective activity of Citronellol was found to be mediated through modulation of prostaglandins (PGE_2_), Cyclooxygenase enzymes (COX-1 and COX-2), 5-Lipoxygenase enzyme (5-LOX), endothelial Nitric oxide Synthase (eNOS), and Intracellular adhesion molecule-1 (ICAM-1) pathways. This study concludes that Citronellol may be a potential therapeutic agent for the treatment of inflammatory condition with prominent gastric protective effects.

## Data Availability

Data will be made available on reasonable request.

## Declaration

### Recommendations

The current study offers a path ahead to investigate the detailed mechanisms of β-Citronellol for the management of inflammatory conditions like Rheumatoid Arthritis, using a targeted approach. This study also highlights the possibility of developing topical Citronellol formulations to reduce arthritis-related pain and inflammation.
